# Synchronous and anti-phase drumming elicit similar prosocial behavior ratings

**DOI:** 10.3389/fcogn.2024.1472814

**Published:** 2024-12-11

**Authors:** Sean McWeeny, Adam C. Luoma, Yaseen Al-Saleem, Laurel J. Trainor

**Affiliations:** ^1^Department of Psychology, Neuroscience, and Behaviour, McMaster University, Hamilton, ON, Canada; ^2^Institute for Music and the Mind, Faculty of Science, McMaster University, Hamilton, ON, Canada

**Keywords:** prosocial behavior, sensorimotor synchronization, Bayesian modeling, music cognition, synchrony

## Abstract

**Purpose:**

Music performance facilitates prosociality across many cultures and contexts. Interestingly, the relationship between prosociality and sensorimotor synchronization (SMS) has so far primarily been demonstrated in the context of in-phase synchrony with only a few mixed results for anti-phase coordination. In anti-phase coordination, participants move at the same rate, at opposite phases, which also requires high levels of coordination and attention. This case is particularly relevant for music and prosociality, as music regularly involves naturalistic anti-phase coordination. We thus tested whether anti-phase synchronization is as effective as in-phase synchronization at eliciting prosocial behavior.

**Methods:**

Dyads (*N* = 50 dyads) were randomly assigned to complete four trials of a drumming sensorimotor synchronization-continuation task (SCT) either alone, synchronously or in anti-phase. Before and after the drumming task, dyads completed a behavioral economics game involving trust. Additionally, a questionnaire about trust, cooperation, affect, and similarity was given after the drumming task.

**Results:**

Cooperation rates in the stag-hunt game were near ceiling (~87%) across all conditions pre-SCT, with negligible change after the drumming task. Questionnaire items were analyzed using Bayesian probit mixed effects models to account for dyadic sampling and ordinal data, and to provide evidence in favor of the null hypothesis. Models provided moderate to extremely strong evidence that the anti-phase and in-phase coordination conditions rated their affect, trust, similarity, and cooperation more strongly than dyads in the alone condition (all BF_10_ > 3). When only comparing the anti-phase and in-phase conditions, moderate evidence in favor of the null (i.e., that phase does not affect ratings) was found for all questions (all BF_10_ < 0.3). Descriptions of the posterior, as well as leave-one-out cross validation (LOO) results, were in general accordance with the Bayes Factor results.

**Conclusion:**

Evidence indicates anti-phase drumming coordination is as effective as in-phase in increasing perceived trust, cooperation, affect, and similarity. Future analyses will examine how other characteristics of the drumming coordination, such as the lag-1 autocorrelation and variability of the inter-tap interval time-series, relate to prosocial behavior and ratings of trust and cooperation.

## 1 Introduction

Music performance facilitates cooperation, trust, and affiliation (i.e., prosocial behaviors) across many cultures and contexts (Savage et al., 2021; Tarr et al., 2014). Due to many universal features of music (Drake and Bertrand, 2001; Jacoby et al., 2019; Savage et al., 2015) and the importance of cooperation in early humans, several evolutionary hypotheses on the origins of music have emerged that explain music's role in social behavior and communication (Mehr et al., 2020; Savage et al., 2021; Schulkin and Raglan, 2014; Wang, 2015). One view by Savage et al. (2021) proposes that music facilitates a wide range of prosocial behaviors (e.g., cooperation, trust, affiliation) through co-activation of highly overlapping dopaminergic networks responsible for social and prediction-based rewards (Atzil et al., 2017; Cheung et al., 2019; Gold et al., 2019). The authors argue that the repeating regularities in music (beat, scales, etc.) provide opportunities to satisfy predictions through movement, and that when paired with social stimuli, reward becomes associated with the communicative partner or group (Fiveash et al., 2023). Regardless of its biological and evolutionary origins, the relationship among music, movement, and social behavior has been clearly demonstrated and is of great interest.

The coordination of rhythmic movement with external rhythms is referred to as sensorimotor synchronization (SMS), and many fundamental properties of SMS have been described (Repp, 2005; Repp and Su, 2013). A special case of SMS, referred to as movement synchrony, defined here as moving or vocalizing at the same rate *and* in-phase with one or more partners, has been shown to increase trust, liking, affiliation and cooperation between people (Mogan et al., 2017; Rennung and Göritz, 2016). Because beat is highly predictable, music is an ideal stimulus to promote synchrony among people as it enables motor planning and action to a steady pulse. Increases in prosocial behavior, often considered a downstream effect of social bonding (though for a disambiguation, see Tarr and Dunbar, 2023), have been demonstrated in infants as young as 14 months after moving in sync with an experimenter (Cirelli et al., 2014a,b; Trainor and Cirelli, 2015). Seemingly regardless of context, increases in synchrony are associated with increases in prosocial behavior and feelings, including trust and cooperation (measured by surveys and economics games; Mogan et al., 2017; Rennung and Göritz, 2016).

Interestingly, the relationship between prosociality and SMS has so far primarily been demonstrated in the context of in-phase synchrony. The relationship between synchrony and prosociality has been tested in numerous contexts, including walking in-step (Wiltermuth and Heath, 2009), large limb movements (Reddish et al., 2013; Exps. 1 and 3), chanting (Reddish et al., 2013; Exp. 2), and singing (Anshel and Kipper, 1988). Synchronous movements seem to induce prosocial behavior above and beyond “coordinated movements” across all synchrony contexts (for a meta-analysis, see Mogan et al., 2017); however, the results of this analysis raise some concerns. In this meta-analysis, coordinated movements included participants moving to their own metronomes at different tempi. Although this technically fits the definition of SMS, as each participant is synchronizing to an external rhythm, it is not coordinated among participants. Temporally “coordinated movements” thus must be further specified before proper conclusions can be drawn.

One type of SMS of particular interest is anti-phase coordination, in which participants move at the same rate, at opposite phases, as would two people on opposite ends of a seesaw. This case is particularly relevant for music and prosociality, as music and dance are perhaps the clearest contexts for naturalistic anti-phase coordination. In-phase and anti-phase sensory events give rise to coupled oscillatory activity in sensory (e.g., auditory) and motor networks, which underlie beat perception and movement to a beat (i.e., entrainment; Large et al., 2015).

There is mixed evidence that anti-phase or alternating (e.g., among a group of 3 participants, not technically anti-phase) SMS among individuals elicits prosocial behavior. Evidence in which anti-phase or alternating SMS failed to elicit prosocial behavior includes studies in which participants alternated chanting words (groups of 3, Reddish et al., 2013; Exp. 2), moved their forearm up and down in anti-phase with an experimenter (Macrae et al., 2008), or tapped alternating in anti-phase (Launay et al., 2013; though anti-phase was not explicitly instructed) with another subject or confederate. The literature describing alternating or anti-phase relationships failing to elicit prosocial effects generally falls into one of three categories: non-naturalistic, non-musical, or individual goal-oriented. Non-naturalistic and non-musical anti-phase examples include each member of a group of 3 chanting one word at a time in an alternating fashion (Reddish et al., 2013; Exp. 2), or moving large limbs in non-naturalistic experimental contexts (Macrae et al., 2008). Individual goal-oriented examples include each participant having their own metronome that they were instructed to follow (e.g., Experiment 3, Reddish et al., 2013) with no attempt to coordinate with another person.

There are three examples of note in which anti-phase coordination has a positive association with prosocial behavior and ratings of prosocial behavior (Cirelli et al., 2014a; Cross et al., 2016; Miles et al., 2009), two of which were non-musical. In Miles et al. (2009), participants rated two stick figures' rapport while walking in various phase relationships. Participants rated both in-phase and anti-phase step coordination as having equally good rapport. This finding is relatively limited given that there was no motor involvement. Better evidence for anti-phase movement eliciting prosocial behavior comes from Cirelli et al. (2014a) and Cross et al. (2016).

In Cirelli et al. (2014b), 14-month-old infants were bounced to music with an experimenter synchronously, asynchronously, and in anti-phase. Infants were significantly more likely to help the experimenter if she “accidentally” dropped an object she was holding in both the synchronous and anti-phase conditions as opposed to the asynchronous condition. In Cross et al. (2016), participants coordinated joystick movement with a partner either in phase, in anti-phase, or asynchronously. Anti-phase movement only elicited increases in cooperation, measured via behavioral economics games (public goods game and an investment game), when the participants saw each other through a mirror, as opposed to only via a point light display (i.e., during increased social context). These studies suggest that anti-phase coordination may be just as effective as in-phase coordination at eliciting prosocial behavior, but the evidence to-date is incomplete.

In the following experiment, we utilized a synchronization-continuation task in which participants either drummed synchronously in-phase, alternatingly in anti-phase, or alone. The anti-phase condition allowed participants to naturalistically create and maintain a shared musical representation with a partner, with the prediction that the in-phase and anti-phase conditions would elicit similar prosocial behavior, as compared to participants completing the task by themselves. Prosocial behavior was measured by a behavioral economics game similar to the prisoner's dilemma, the stag-hunt game (e.g., Fang et al., 2002). In addition, participants rated their feelings of trust, cooperation, and affect both generally and as they related to task-performance (i.e., prosocial behavior ratings). The chosen outcomes, a behavioral economics game and a questionnaire on synchronization performance and trust, have been successfully utilized in prior research (Reddish et al., 2013).

## 2 Methods

### 2.1 Participants

Students attending university (*n* = 106, 20 males, age range 17–26) voluntarily participated in the experiment. Participants were randomly assigned to dyads (*N* = 53). Each dyad was quasi-randomly assigned to a condition for the synchronization-continuation task: Alone (*N*_Alone_ = 10), In-Phase (*N*_I−P_ = 22), or Anti-Phase (*N*_A−P_ = 21). Fewer dyads were collected for the Alone condition, as it quickly became clear that sufficient data had been collected to meet the study aims. Dyads were asked if they had a conversation with their assigned partner prior to the experiment to ensure dyads were made up of strangers with no prior social interaction. Subjects were also instructed not to speak or interact with their partner before the study took place. Participants were recruited through a university research participation portal and did not need to have music experience to participate, though 43% had at least 5 years of formal music education. The study was approved by the McMaster Research Ethics Board, and all subjects gave written consent agreeing to the experimental procedure.

Three dyads (*N*_I−P_ = 1; *N*_A−P_ = 2) were excluded from all analyses; one of which was excluded due to a procedural error in the order of events, and two of which were excluded due to social interaction before testing. Of the remaining participants, *n* = 32 played a musical instrument at the time of the study, *n* = 30 had five or more years of formal musical education, and *n* = 15 had no formal musical education. They were compensated with course credit as well as payment within a range of $0–$10 based on the results of the stag-hunt game.

### 2.2 Procedure

**Figure 1:**
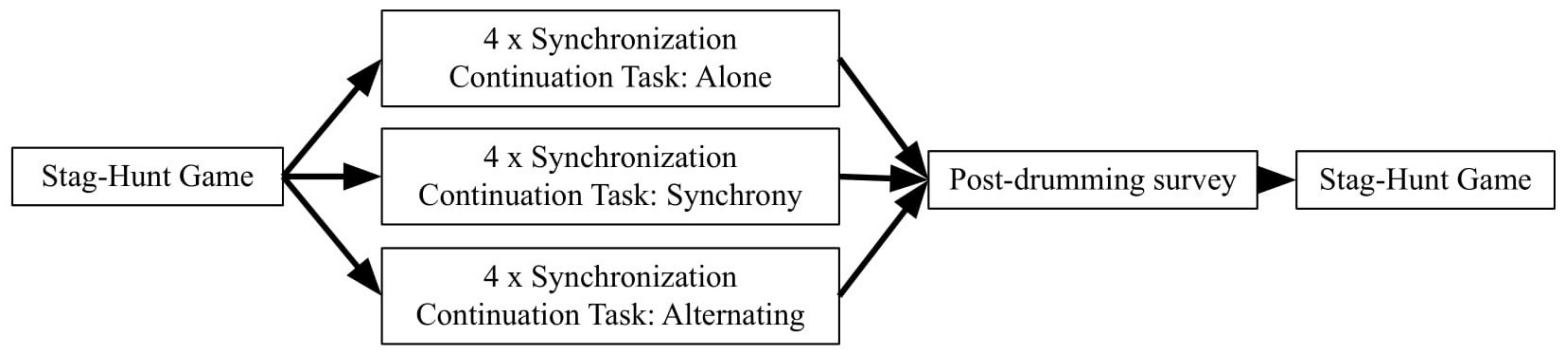
Experimental design flow for all participants.

Condition was randomly determined prior to participant arrival. Upon arrival, participants filled out demographics forms and were given a number for random assignment to their dyad partner. Once the dyad had completed the demographics form, they completed one round of the stag-hunt game, then four trials of a synchronization-continuation task, followed by a questionnaire, and finally, another round of the stag-hunt game before being debriefed. This procedure is shown in Figure 1.

#### 2.2.1 Setup and drumming apparatus

For the Anti-Phase and In-Phase conditions, participants sat in chairs ~2 m across from each other in front of an electronic drum pad. In the alone condition, they were seated in different rooms, and white noise was played to ensure sound isolation from each other's drum noises. A line of sight was available when certain doors were open, allowing instructions to be given by the experimenter simultaneously to both participants between trials.

Participants used a wooden drumstick to drum on a Yamaha TP70 electronic drum pad, connected to a Yamaha DTX900 sound module. The digital audio workstation REAPER was used to collect MIDI data from the drum pads and to send synchronization triggers to the drum module for stimulus timing. Each participant had their own speaker placed on the floor beside them at 90° that would produce their drum's sound.

#### 2.2.2 Synchronization-continuation task

Each dyad completed four trials of a synchronization-continuation drumming task. Each participant's drum played a tom sound separated by a major 3^rd^, which was chosen to balance having each drummer having their own clearly identifiable sound, while still making the sounds naturalistic. This assignment switched between each participant on each trial.

All three conditions involved a synchronization phase of four beats for eight measures and a continuation phase of 60 seconds. The synchronization stimulus, a kick drum on beats 1 & 3, and a snare drum on beats 2 & 4, was played (a) on a third speaker placed between the participants for the In-Phase and Anti-Phase conditions or (b) on the single speaker placed by each participant in their separate rooms for the Alone condition. For the Alone condition, participants could only hear the sounds resulting from their synchronization stimulus and their own drum hits due to sound isolation between the rooms.

For the In-Phase and Alone conditions, the synchronization stimulus was played at 60 bpm. During the synchronization phase, both participants were instructed to drum along to both the kick and snare drums, resulting in a perceived tempo of 60 bpm with a 1 Hz motor tempo. For the Anti-Phase condition, the tempo was doubled (120 bpm), but the participant with the lower-pitched tom was instructed to synchronize with the kick sound (beats 1 & 3), and the participant with the higher-pitched tom was instructed to drum along with the snare sound (beats 2 & 4). As such, the motor tempo was held steady at 1 Hz across conditions. This was done to control for exertion, as prior research has found that exertion plays a role in the relationship between synchronization and social bonding (Sullivan and Blacker, 2017; Tarr et al., 2014).

Participants in all conditions were instructed that after some time, the kick-snare metronome would stop, and they were to continue drumming in the same manner as the synchronization phase as steadily as possible. The continuation phase lasted 60 seconds and was ended by a rapid drum sound that would interrupt the participants. If participants needed an additional prompt to continue after the synchronization phase, they were given the instruction between trials to continue drumming as steadily as possible. Participants were not allowed to talk between trials while the experimenter changed which participant had which pitched drum (i.e., high or low).

#### 2.2.3 Prosocial behavioral measures

To evaluate prosocial behavior, participants played a stag-hunt game and completed a questionnaire as in Reddish et al. (2013), Exp. 2. The stag-hunt game is similar to the prisoner's dilemma, but in the prisoner's dilemma, the only pure-strategy (i.e., Nash equilibria) is to defect, whereas in the stag-hunt game, cooperation is also a pure-strategy (Fang et al., 2002). As such, strategy depends on what each participant thinks the other is doing more than in the prisoner's dilemma. For the game, participants were given task instructions detailing a scenario in which attempting to work together for a group project would be risky, but could result in a larger reward if the other participant agreed to work together (see full task instructions on our Open Science Framework (OSF) page, linked at the end of the manuscript). The participants were asked to circle either “Work Alone” or “Work Together,” and the latter option is referred to as “cooperated” throughout the manuscript. The game was played twice, once after demographic information was collected and once after the four trials of the drumming task. The participants were not made aware of the outcome of either game until the end of the experiment, and were instructed not to interact during the game. To further incentivize participants, they were told they could win $0–$10 during the study. Their performance in the game directly translated to money won. Each A was worth $5, each B was worth $2.50, and each C was worth $0. Table 1 displays the four different payout results.

**Table 1:** Payout structure for the stag-hunt game.

	**Participant 1 works alone**	**Participant 1 works together**
**Participant 2 works alone**	Both get $2.50	P1 gets $0 P2 gets $2.50
**Participant 2 works together**	P1 gets $2.50 P2 gets $0	Both get $5

Six 7-point Likert-type questions were also used to assess participants' trust, cooperation, affect, and perceived similarity, the first five of which were taken or adapted from Reddish et al. (2013; Experiments 1–2). These questions are presented in Table 2. “Happy” was not assessed in Reddish et al. (2013), but positive affect has been studied in relation to synchronization, though with quite broad definitions (for a meta-analysis, see Mogan et al., 2017; Fessler and Holbrook, 2014; Lumsden et al., 2014).

**Table 2:** Questionnaire items assessed post-drumming.

**Shorthand**	**Full question (1–7 Likert)**
Unit	How much did you feel you and the other participant were a unit?
Same Team	How much did you feel you were on the same team with the other participant?
Trust Before	How much did you trust the other participant going into the exercise?
Similar	How similar are you to the other participant?
Cooperated	How much did you feel you and the other participant cooperated during the task?
Happy	How happy are you right now?

### 2.3 Statistical approach and analysis plan

Our initial plan for the stag-hunt game analysis was to run a mixed chi-square test with the between-subjects factor of condition and within-subjects factor of pre- and post-drumming; however, as will be shown in the results, given characteristics of the data, this was not ideal for several reasons. We thus shifted the focus of our analysis to the questionnaire items.

**Figure 2:**
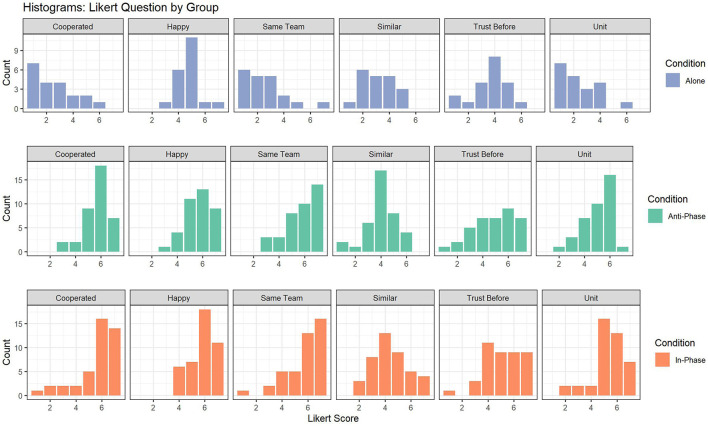
Histograms for each likert question and group.

Our two research questions were (a) whether the In-Phase and Anti-Phase conditions yielded higher ratings than the alone condition on the questionnaire items (largely a manipulation check) and (b) whether the In-Phase and Anti-Phase conditions yielded similar ratings on the questionnaire items (our main question). For both research questions, we utilized Bayesian probit mixed effects models with *brms* in R (version 2.20.4, Bürkner, 2017; Stan version 2.26.1). The choice of mixed effects models and a probit link function was motivated by dyadic sampling and responses that were highly skewed and ordinal by nature (Figure 2), respectively.

The rationale for choosing Bayesian models was manifold; primarily, we wanted to be able to compare evidence among our hypotheses, namely the null hypothesis that in-phase and anti-phase coordination are equivalent in their effects on prosocial behavior ratings. Frequentist statistics only allow for a failure to reject the null, rather than provide evidence for or against it, which is crucial in the In-Phase vs. Anti-Phase analysis. For consistency, the same models were chosen for the analysis comparing the Alone condition to the Anti-Phase and In-Phase conditions.

As the six outcome variables were ordinal and highly skewed, particularly when only comparing Anti-Phase vs. In-Phase (Figure 2; Histograms), we utilized mixed cumulative probit regression. The primary motivation for using ordinal regression was due to the differences in the cumulative distribution functions and histograms for the different questions. For example, participants were much more likely to mark a 7-point response for “Same Team” as they were for “Unit,” despite both questions being task-performance related. Below, we provided context for the parameters of the probit models and how they map to the 7-point ordinal scale in explicit terms to aid interpretation. In addition, Gaussian alternatives were generally in agreement with those generated by the probit models with the exception for “Cooperated.”

#### 2.3.1 Priors and model specification

All parameters were specified in log-odds units. A random intercept term, σ, was included to account for dyadic sampling in the null and full models. Condition (In-phase, Anti-phase, and Alone) was included as a predictor in the full models for the two research questions. For all models used in both research questions, the σ prior was the default half Student's *T*(0, 2.5, df = 3). The priors for each Θ intercept were specified 0.5 SD away from each other with an SD = 2 [e.g., starting at Normal (−1.5, 2) and ending at Normal (1, 2) for each Θ]. For several questions, only *k* = 5 or *k* = 6 (of 7) response categories were used, requiring that the data be rescaled serially from 1 to *k*. Small modifications were made to allow the models to converge; full prior specification is available on our OSF page. The Θ parameters are not of interest for our research question, though they can aid in interpretation of ß, the parameter of interest.

The two research questions (i.e., In-Phase and Anti-Phase vs. Alone, and In-Phase vs. Anti-Phase), required the use of different priors. All priors were generated using effect size benchmarks that were converted from Cohen's *d* to log-odds ratios. This conversion is done by multiplying the commonly used Cohen's *d* cutoffs of *d* = 0.2, *d* = 0.5, and *d* = 0.8 for small, medium, and large effects, respectively, by 1.814 (Hasselblad and Hedges, 1995; small log-OR = 0.36; medium log-OR = 0.91; large log-OR = 1.45). For analyses including the Alone condition data, a Normal (1,2) ß prior was used for condition. A Normal (1, 2) prior contains 13% of its probability mass as no effect (i.e., smaller than a small effect), 18% of its probability mass as a small effect, 45% as a medium effect, and 24% as a large effect or larger. For analyses only including the Anti-Phase and In-Phase conditions, a Normal (0, 2) ß prior was used. A Normal (0, 2) prior contains 14% of its probability mass as no effect (i.e., smaller than a small effect), 21% of its probability mass as a small effect, 46% as a medium effect, and 19% as a large effect or larger. The justification for centering the prior at *M* = 1 for the models including the Alone condition is that we expect large effects for more different conditions (drumming together vs. alone, as compared to drumming together in-phase or anti-phase).

As these types of models may be unfamiliar to readers, it is worth explaining how to interpret the parameter values. For example, Θ = −1.0 would indicate that *p* = 0.27 of the responses fall below that Θ, and a ß = 0.7 would indicate that beingin a given condition (e.g., In-Phase) would increase the log-odds by 0.7 compared to the comparison condition (e.g., Alone). The mapping to the 7-point scale is thus done by the Θ values, as a 0.7 increase in log-odds is more easily interpreted if we see that the distance between two Θ values is, for example, 0.7 log-odds units (i.e., one scale value). The log-odds value can be characterized as an odds ratio (OR), by exponentiating the value, which may help with effect size interpretation; however, the intuitive interpretation of the OR breaks down when the outcome is not dichotomous.

Prior predictive checks were examined using empirical cumulative distribution function (ECDF) plots and the traces seemed reasonable, though slightly skewed as compared to the data, as is expected from the data histograms. Full priors and prior predictive checks for each model are presented at our OSF page.

#### 2.3.2 Bayes Factors

Bayes Factors were calculated using the *bayestestR* package in *R* (Makowski et al., 2019). We calculated the Savage-Dickey Density Ratio (Wagenmakers et al., 2010) of the posterior parameter for condition compared to a Normal (0,2) prior. Bayes Factors using the condition model compared to the null model were also calculated and provided nearly identical values to the Savage-Dickey Density Ratio.

#### 2.3.3 Model comparison

In addition to Bayes Factors, we compared the random intercept-only model to the model with condition as a predictor. The purpose of this analysis was to provide complementary information and mitigate the risk of overfitting by using leave-one-out cross validation (LOO). We did this by using *brms::loo*, which calculates the expected log pointwise predictive density (ELPD). If the Pareto *k* of any point was >0.7, the *loo* model was recomputed using moment_match = T, and none of our final LOO models had any Pareto *k* > 0.7.

## 3 Results

### 3.1 Relationships among prosocial behavior measures

Cooperation rates for the stag-hunt game for all conditions were near ceiling, both before and after the synchronization-continuation task. The cooperation rates for each condition, before and after the synchronization-continuation task, are presented in Table 3. We thus shifted the remainder of our analyses to use the questionnaire items. We were also interested in the relationships among the items, whose means and SDs are presented in Table 4 and whose distributions are presented in Figure 2. We therefore calculated Spearman correlations among the items which are shown on our OSF page and demonstrate a range of positive correlations, with stronger correlations among the task-relevant items.

**Table 3:** Contingency table for stag-hunt game.

	**Alone**	**Anti-phase**	**In-phase**
Pre	17/20	32/38	39/42
Post	17/20	33/38	37/42

Participants cooperated/total participants.

**Table 4:** Means and SDs for each Likert question treated continuously.

	**Alone**	**Anti-phase**	**In-phase**
Unit	2.40 (1.20)	5.05 (0.85)	5.36 (0.95)
Same Team	2.55 (1.17)	5.76 (0.63)	5.76 (1.00)
Trust Before	3.70 (0.82)	4.89 (0.91)	5.14 (1.06)
Similar	3.15 (0.85)	4.07 (0.78)	4.40 (1.02)
Cooperated	2.55 (1.09)	5.68 (0.67)	5.67 (1.26)
Happy	4.75 (0.42)	5.66 (0.71)	5.81 (0.70)

### 3.2 Prosocial behavior across conditions

#### 3.2.1 Alone, anti-phase and in-phase conditions

The full posterior for each item is reported on our OSF page. Model results for the ß parameters for each item are presented in Table 5. The items fall roughly into two categories: explicitly task-related questions (“Unit,” “Same Team,” and “Cooperated”) and non-task-related questions (“Trust Before,” “Similar,” and “Happy”). Unsurprisingly, the explicitly task-related questions have extremely large effects (e.g., from OR = 8.85 to OR = 13.46; from ß = 2.18 to ß = 2.60), as the comparison is to the Alone condition. More interestingly, the non-task-related questions show more modest, but still considerable effects (from OR = 2.18 to OR = 3.13; from ß = 0.78 to ß = 1.14). LOO results also generally broke down along these lines, with explicitly task-related questions being at least 1.75 SE improvement in ELPD (i.e., model fit), with non-task-related questions generally falling closer to 1 SE improvement in ELPD. LOO results are presented in Table 6.

**Table 5:** Regression weights for in- and anti-phase coordination vs. alone.

	**Anti-phase**			**In-phase**		
**Question**	***ß***	**Error**	**95% HDPI**	***ß***	**Error**	**95% HDPI**
Unit	2.23	0.45	[1.39 3.17]	2.60	0.46	[1.76 3.55]
Same Team	2.18	0.36	[1.49 2.90]	2.20	0.36	[1.51 2.92]
Trust Before	0.86	0.31	[0.26 1.47]	1.05	0.30	[0.45 1.65]
Similar	0.78	0.32	[0.16 1.43]	1.09	0.32	[0.48 1.73]
Cooperated	2.21	0.44	[1.41 3.11]	2.39	0.44	[1.59 3.30]
Happy	0.97	0.32	[0.36 1.60]	1.14	0.32	[0.53 1.77]

**Table 6:** ELPD differences, BF, and posterior parameter ß—alone vs. anti-phase and in-phase conditions.

**Normal (1,2) Prior**					
**Question**	**ELPD Diff (SE)**	**BF**_10, A − P_	**BF**_10, I − P_	_A − P_	_I − P_
Unit	9.7 (4.3)	12,700	627,000	2.23	2.60
Same Team	17.5 (5.8)	3,760,000	5,180,000	2.18	2.20
Trust Before	4.0 (3.0)	8.22	56.93	0.86	1.05
Similar	3.7 (3.1)	3.50	64.68	0.78	1.09
Cooperated	8.3 (4.7)	34,200	52,400	2.21	2.39
Happy	0.1 (0.0)	21.29	106.15	0.97	1.14

Bayes Factors on the explicitly task-related questions all showed extremely strong evidence in favor of both the Anti-Phase and In-Phase drumming conditions as compared to the Alone condition (all BF_10_ ≥ 30,000). For non-task-related questions, evidence ranged from moderate to extreme in favor of both the Anti-Phase and In-Phase drumming conditions: “Trust Before” (BF_10, Anti−Phase_ = 8.22; BF_10, In−Phase_ = 56.93), “Similar” (BF_10, Anti−Phase_ = 3.50; BF_10, In−Phase_ = 64.68), and “Happy” (BF_10, Anti−Phase_ = 21.29; BF_10, In−Phase_ = 106.15). Robustness analyses detailing alternative priors are included in on our OSF page.

#### 3.2.2 Anti-phase vs. in-phase conditions

To directly test whether there were differences between the Anti-Phase and In-Phase conditions, we excluded the data from the Alone condition. The resulting models and posteriors for each item are reported in full on our OSF page. Estimations of the ß for each question are reported in Table 7. Interestingly, unlike in the Alone condition analyses, the ß parameters do not group by explicitly- vs. non-task-related; however, for parallel structure and clarity, we will maintain this distinction. ßs were generally ≤ 1 SE, with “Unit” and “Similar” just barely crossing that threshold. LOO results indicated worse model fit for the models containing condition as a predictor (Table 8).

**Table 7:** Posterior ß for anti- vs. in-phase coordination.

**Question**	**ß**	**Error**	**95% HDPI**
Unit	0.35	0.31	[−0.25 0.97]
Same Team	0.02	0.26	[−0.48 0.53]
Trust Before	0.18	0.25	[−0.31 0.68]
Similar	0.30	0.26	[−0.21 0.82]
Cooperated	0.18	0.34	[−0.49 0.87]
Happy	0.17	0.26	[−0.35 0.68]

**Table 8:** Posterior ß for anti- vs. in-phase coordination.

	**Normal(0,2)** ***ß*** **Prior**			**Normal(0,1)** ***ß*** **Prior**		
**Question**	**ELPD Diff (SE)**	**BF**_10_	***ß***	**ELPD Diff (SE)**	**BF**_10_	***ß***
Unit	−0.1 (1.1)	0.29	0.35	−0.1 (1.0)	0.56	0.33
Same Team	−1.3 (0.2)	0.13	0.02	−1.2 (0.2)	0.25	0.02
Trust Before	−0.9 (0.8)	0.16	0.18	−0.9 (0.7)	0.31	0.17
Similar	−0.4 (1.1)	0.25	0.30	−0.4 (1.0)	0.46	0.28
Cooperated	−1.1 (0.7)	0.19	0.18	−0.7 (0.5)	0.35	0.16
Happy	−1.1 (0.6)	0.16	0.17	−0.9 (0.6)	0.30	0.16

The BF_10_ for each full model was calculated against a null model (i.e., the respective prior used by each model).

For all the items, there was moderate evidence that the anti-phase and synchrony conditions did not differ on “Unit” (BF = 0.29), “Same Team” (BF = 0.13), “Trust Before” (BF = 0.16), “Similar” (BF = 0.25), “Cooperated” (BF = 0.19), and “Happy” (BF = 0.16). The cutoff value of BF ≤ 0.33 for moderate evidence for the null is simplistic by design, so it is useful to interpret the values themselves. For example, there is 7.7 times (1/0.13) more evidence for the null hypothesis that “Same Team” did not differ between synchrony and anti-phase conditions.

##### 3.2.2.1 Robustness analyses

Using a narrower [i.e., Normal (0,1)] ß prior, the models were refit. The posterior ß of the narrow prior had excellent agreement with the original model, always differing by < 0.02, shown in Table 7. The LOO analyses also showed excellent agreement, with the ELPD difference and its SE changing by < 0.2 except for “Cooperated,” for which the ELPD difference changed by 0.4 and its SE changed by 0.2.

For Bayes Factor analyses, the general trends stayed the same, but the specific values changed approximately by a factor of 2 using the narrower prior. Thus, using the narrow prior, there was anecdotal to moderate evidence that the anti-phase and in-phase conditions did not differ on “Unit” (BF_10_ = 0.56), “Same Team” (BF_10_ = 0.25), “Trust Before” (BF_10_ = 0.31), “Similar” (BF_10_ = 0.46), “Cooperated” (BF_10_ = 0.35), and “Happy” (BF_10_ = 0.30).

## 4 Discussion

In the current study, we tested two predictions about the prosocial effects of coordinating a drumming task with a partner. We did this by randomly assigning dyads to perform a synchronization-continuation drumming task either in-phase, anti-phase, or alone, and measuring prosocial behavior with a behavioral economics game and a questionnaire. The behavioral economics stag-hunt game yielded extremely high baseline levels of cooperation, with nearly identical cooperation rates across time (i.e., pre- and post-drumming) and condition, leading us to focus on the questionnaire. Using the questionnaire items, we first tested whether drumming either in-phase or anti-phase yielded higher levels of trust, cooperation, affect, and perceived similarity, as compared to drumming alone. Results provided moderate to very strong evidence that drumming with a partner increased ratings, including for non-task-related questions such as “how similar are you to the other participant,” “how happy are you right now,” and “how much did you trust the other participant going into the exercise.” Next, we tested the extent to which the Anti-Phase and In-Phase conditions yielded the same trust, cooperation, affect, and perceived similarity ratings. The Bayesian models provided moderate evidence *in favor of the null hypothesis* that these conditions yielded the same prosocial effects. Analyses of the posterior, LOO, and Bayes Factor results were generally in concordance, with some evidence of overfit indicated by LOO results where the full model had worse fit (i.e., ELPD) despite a ß ≥ 1 SE (e.g., “Unit” in Table 8).

Our results were relatively robust, though some concern is relevant for the Bayes Factor interpretations, as some questions (e.g., “Unit” in In-Phase vs. Anti-Phase) changed categories, though generally remaining anecdotal-to-moderate when using a different prior comparison. This is a well-documented problem with Bayes Factors, and a one of many motivations to running robustness analyses (Kruschke, 2021). Nonetheless, the posterior and LOO analyses showed relatively unequivocal results regardless of the chosen prior. The Bayes Factors should thus (a) be considered in their raw values (i.e., there is BF_10_ times more evidence for model 1 than model 0) and (b) be cautiously interpreted, using LOO and posterior results to support the conclusions.

### 4.1 Meta-analytic and effect size contextualization

#### 4.1.1 Alone condition vs. anti-phase and in-phase

Contextualizing these results is a challenge due to the wide variation in control tasks used across the literature. Nonetheless, our primary source for such comparisons is drawn from the Mogan et al. (2017) meta-analysis. Their control conditions fell under two categories: “socially coordinated” or “no action”; our Alone condition would fall neatly under their “no action” category, whereas our Anti-Phase condition would fall under the socially coordinated category. As compared to the no action controls, synchronous movement resulted in small effects on perception and affect (*d* < 0.2). Here, despite including one of their control conditions as one of our experimental conditions (i.e., “socially coordinated” and Anti-Phase), we found medium effects for positive affect (“Happy” log-OR = 0.97–1.14), similarity (log-OR = 0.78–1.09), and trust (log-OR = 0.86–1.05), whereas we found extremely large effects for cooperation perception (“Unit,” “Same Team,” and “Cooperated” log-OR = 2.18–2.60). We purport that the present study would have met inclusion criteria for this meta-analysis and may have skewed results upwards due to the solitary nature of our Alone condition. Control tasks may need to be more carefully grouped if meaningful effect sizes are meant to be derived from such a meta-analysis, and the results presented here should not be thought to extend to interactive control tasks.

#### 4.1.2 Anti-phase vs. in-phase

Contextualizing the In-Phase vs. Anti-Phase results is a much more straightforward task due to the much smaller set of studies (Cirelli et al., 2014a; Cross et al., 2016; Launay et al., 2013; Macrae et al., 2008; Miles et al., 2009; Reddish et al., 2013, though Reddish 2013 Exp. 2 would be better described as phase-opposition). Though not every study of this group reports an effect size, estimates range from no effect (Cirelli et al., 2014a; *F*_1,40_ = 0.14; η^2^_*p*_ = 0.003) to medium [Reddish et al., 2013 Exp. 2; $\chi_{\left(1,27\right)}^{2}$ = 4.49; *V* = 0.41] to large (Macrae et al., 2008; *p*_1_ = 0.8, *p*_2_ = 0.4; *h* = 0.85), which helped motivate our choice in broad priors. As the posterior ßs for all questions were (a) smaller than a small log-OR and generally ≤ 1 SE, the LOO fit decreased when adding condition as a predictor, and the Bayes Factors generally provided moderate evidence in favor of the null, our conclusions can be robust. The present results thus provide further evidence that in-phase and anti-phase coordination do not differ on their effects on prosocial behavior ratings of trust, cooperation, affect, and perceived similarity.

#### 4.1.3 Contributions to the literature

The present study contributes to theoretical perspectives that posit causal pathways among music, synchronization, and prosocial behavior (Demos et al., 2012; Launay et al., 2016; Tarr et al., 2014). Specifically, this study further deemphasizes the role of identicality in self-other merging (also seen as self-other blurring or blending; Decety and Sommerville, 2003; Launay et al., 2016). Though we did not measure self-other merging in this study, as we did not want to prime our participants too heavily before the stag-hunt game, hypotheses would generally predict much stronger self-other merging in the In-Phase condition. Given that our prosocial behavior ratings remained equivalent across In- and Anti-phase conditions, understanding the relationship between phase and self-other merging will thus be a key question to address in future studies.

There are two primary methodological strengths of the present study that merit further discussion: eliciting naturalistic synchronization behavior and statistical modeling. In contrast to prior literature, our coordination task was naturalistic and musical. Prior literature has used non-naturalistic, non-musical stimuli or tasks such as rating point-light displays (Miles et al., 2009), alternating chanting words among a group of 3 (Reddish et al., 2013; Experiment 2), or walking in-step (Wiltermuth and Heath, 2009). Despite the sample being primarily non-musicians, participants were generally successful completing the task, striking a balance as a plausibly naturalistic musical task for that could be completed by musicians and non-musicians alike.

The other strength of our approach lies in the details of the Bayesian models. First, the use of mixed effects ordered probit regression allowed us to model the ordinal and dyadically sampled nature of the data, as opposed to treating items as continuous variables and using parametric or non-parametric frequentist statistics (e.g., Reddish et al., 2013). Though interpretation can be a challenge for ordered probit models, we have provided the reader with a mathematical interpretation of the log-odds ß and the distance between Θ intercepts, as well as the size of these effects in practical terms. Secondly, the use of Bayesian models has allowed us to provide evidence in favor the null hypothesis, as well as evaluate the robustness of these claims under different priors. Though the Bayes Factor results highly depend on the choice of prior, as it is directly used in the analysis, the posterior description and LOO and results are robust to prior choice.

Though less emphasized here, due to the nature of the stag-hunt game results, this study was the first to measure prosociality before and after synchrony; this allowed us to measure baseline rates of cooperation in our sample, which was much higher than our pilot data suggested. Previous studies have only measured post-synchrony prosociality, and claims relating to synchrony *causing* an increase in prosociality are made on a between-subjects basis, rather than a within-subjects basis. We recommend caution regarding within-subjects claims (e.g., synchrony causes an increase in cooperation) from a between-subjects design (e.g., synchrony condition resulted in higher prosociality than other condition).

Though the stag-hunt game did seem to demonstrate a ceiling effect, the underlying variable of prosociality was still likely captured by the stag-hunt game but was dichotomized at a suboptimal point. Manipulating the payout matrix to encourage working alone and discourage working together should result in lower cooperation rates.

### 4.2 Limitations and future directions

The most obvious concern is the failure of the prosocial behavior task (e.g., stag-hunt) to disambiguate the Alone condition from the In-Phase and Anti-Phase conditions due to ceiling effects. This instead forced us to rely on questionnaire items indexing prosociality. Though in many cases, ratings of prosocial behavior or social bonding correspond highly with behavior (Reddish et al., 2013), there have also been reports in which increased prosocial ratings fail to lead to prosocial behavior (Dunbar et al., 2021; Tarr and Dunbar, 2023). As a goal of the present study was to shift synchrony paradigms toward a more naturalistic scenario, the lack of differences in prosocial behavior reduces the generalizability of these findings. In addition, the questionnaire was relatively limited in scope and had no reverse-coded items. Future research should expand the range of questions to ensure that the increased responses seen here were specific to prosociality and positive affect as opposed to a broader range of outcomes including stress, arousal, or self-confidence.

The other chief concern about our experimental design relates to differences inherent to anti-phase vs. in-phase synchronization. Either the motor tempo or the beat tempo can be equal across the conditions, but not both at the same time; in our experiment, we chose to control the motor tempo, meaning that the beat was twice as fast for the Anti-Phase condition than for the In-Phase condition, though all participants drummed at 1 Hz. This decision was motivated by several factors. First, exertion plays a key role in the relationship between synchrony and prosociality (Launay et al., 2016; Tarr et al., 2014). Though the degree of exertion is much greater in some of the studies linking exertion, synchrony, and the endogenous opioid system (e.g., rowing; Sullivan and Rickers, 2013; Sullivan et al., 2014), there is evidence that anti-phase, non-exertive drumming at 65 BPM can increase pain thresholds (Sullivan and Blacker, 2017). To not exacerbate this potential confound, and to compare with prior literature, we chose to hold motor tempo constant across conditions. Nonetheless, the Anti-Phase condition was perceived at 120 bpm, which may be a more comfortable perceived tempo than 60 bpm. As such, task difficulty may have influenced the results, such that the Anti-Phase condition would have seen boosts in questions relating to task performance (e.g., “Unit”). In addition to controlling for beat tempo and adding other control conditions, additional analyses from the present dataset could test the relationship among SMS-derived variables, such as lag-1 autocorrelation (AC1) or circular variance CV, demographic variables (e.g., musicianship), and ratings of trust, cooperation, affect, and perceived similarity. This approach was previously taken by Hove and Risen (2009), who found that more accurate drumming, as indexed by CV, was related to increased feelings of social affinity and connection. However, due to the already complex nature of our analyses, we opted not to include these analyses here, but we encourage readers to explore the published SMS dataset on our OSF page. Preliminary analyses did not reveal an effect of musicianship, though it is likely a complex relationship between dyad members' experiences (e.g., one musician and one poor synchronizer).

## 5 Conclusion

The current study replicates and extends prior findings that coordinated movements result in increased ratings of prosocial behavior compared to an alone task, regardless of whether the synchronization is in-phase or in anti-phase. Additionally, we found evidence in favor of the null hypothesis that there was no difference in prosocial behavior ratings between in-phase and anti-phase drumming.

## Data Availability

The datasets presented in this study can be found in online repositories. The names of the repository/repositories and accession number(s) can be found below: https://osf.io/aew7q/.
